# CannChange: a protocol for a feasibility study using fMRI-based neurofeedback to change the neurobiology of craving in cannabis use disorder

**DOI:** 10.1136/bmjopen-2025-105854

**Published:** 2025-08-31

**Authors:** Ethan Murphy, Amir Hossein Dakhili, Saampras Ganesan, Andrew Zalesky, Rebecca Glarin, Hannah Thomson, Anastasia Paloubis, Sunjeev K Kamboj, Bradford A Moffat, Govinda Poudel, Chao Suo, Valentina Lorenzetti

**Affiliations:** 1 Neuroscience of Addiction and Mental Health Program, Healthy Brain and Mind Research Centre, School of Behavioral and Health Sciences, Faculty of Health, Australian Catholic University, Fitzroy, Victoria, Australia; 2 Department of Psychiatry, The University of Melbourne, Melbourne, Victoria, Australia; 3 Department of Biomedical Engineering, The University of Melbourne, Melbourne, Victoria, Australia; 4 Contemplative Studies Centre, Melbourne School of Psychological Sciences, The University of Melbourne, Melbourne, Victoria, Australia; 5 Centre for Youth Mental Health, Melbourne Medical School, The University of Melbourne, Melbourne, Victoria, Australia; 6 Department of Psychiatry, Melbourne Medical School, University of Melbourne, Melbourne, Victoria, Australia; 7 Department of Biomedical Engineering, Melbourne Medical School, University of Melbourne, Melbourne, Victoria, Australia; 8 Melbourne Node of the National Imaging Facility, Department of Radiology, The University of Melbourne, Melbourne, Victoria, Australia; 9 Clinical Psychopharmacology Unit, Research Department of Clinical, Educational and Health Psychology, University College London, London, UK; 10 Mary MacKillop Institute of Health Research, Australian Catholic University, Melbourne, Victoria, Australia; 11 BrainPark, Turner Institute for Brain and Mental Health, Monash University, Clayton, Victoria, Australia

**Keywords:** Neurofeedback, Magnetic Resonance Imaging, Cannabis, Craving, Brain

## Abstract

**Abstract:**

**Introduction:**

Cannabis use disorder (CUD) affects ∼33 million people globally and can be underscored by intense cravings to use cannabis, which can trigger compulsive use and relapse. Functional MRI (fMRI) evidence demonstrates hyperactivity of addiction brain pathways during cannabis cue-reactivity, consistent with prominent neuroscientific theories of addiction, particularly within the anterior cingulate cortex (ACC). The ACC also emerges as a key region of real-time fMRI-based neurofeedback (fMRI-neurofeedback) studies demonstrating voluntary changes during cravings in persons who use substances. However, this notion is untested in CUD.

**Methods and analysis:**

We aim to develop a protocol that tests the feasibility of fMRI-neurofeedback to enable persons with a moderate-to-severe CUD to increase the activity of the ACC during cannabis-induced craving to provide mechanistic insights on treatment targets; and decrease ACC activity during cue-induced craving to pave the way for reducing brain reactivity. The primary outcome measure is the change in ACC activity during fMRI-neurofeedback compared with a non-regulation condition.

**Ethics and dissemination:**

This feasibility study has been approved by the Human Research Ethics Committee of Australian Catholic University. On completion, the findings from this study will be published in academic journals, presented at conferences and disseminated to clinicians and to individuals who use cannabis. The results from this feasibility study have the potential to inform the conduct of powered trials to examine how fMRI-neurofeedback can identify and reduce craving-related brain dysfunction in CUD.

**STRENGTHS AND LIMITATIONS OF THIS STUDY**

- This is the first protocol for the use of functional MRI (fMRI)-neurofeedback in individuals who use cannabis.
- The brain target to be used during fMRI-neurofeedback will be individualised, based on the activation within craving-related neurocircuitry during a cannabis cue-reactivity task. This individualised approach to target selection could enable participants to learn to voluntarily regulate in real-time their own specific craving-related brain activity.
- This protocol is limited by the lack of an active placebo control group, transfer runs (ie, where the same task is presented in the absence of feedback) and behavioural follow-ups.

## Introduction

### Cannabis use disorder

 Cannabis use disorder (CUD) affects approximately 33 million people globally.^1 2^ From 1990 to 2019, globally, there has been a 32% increase in the incidence of CUD (from 2 825 090 to 3 737 240) and a 39% increase (from 498 050 to 690 340) in CUD-related disability-adjusted life years due to associated adverse outcomes, such as accidental Δ(9)-Tetrahydrocannabinol (THC) poisoning, anxiety and depression.^3^ Similarly, hospitalisation for CUD is associated with an almost three times higher risk of mortality compared with age and sex-matched non-hospitalised members of the general population.^4^ CUD is associated with several significant adverse psychosocial outcomes, such as unsuccessful attempts to reduce or quit using cannabis, and comorbidity with, and elevated symptoms of anxiety, depression and psychotic disorders.^5^ Current rates of abstinence from cannabis remain modest, and people with a CUD often report difficulty cutting down or quitting their use.^6^,^9^ Further, due to a multitude of barriers,^10^ only 13% of people who experience CUD are estimated to look for treatment over their lifetime.^11^

One characteristic contributing to the compulsive and chronic use of cannabis in CUD is the experience of strong craving, defined as the intense desire and preoccupation to use cannabis.^12^ Craving has been found to intensify following the presentation of cannabis-related cues (eg, advertisements of cannabis and paraphernalia).^13^ With the international trends towards increased decriminalisation of cannabis products,^14^ environmental cues of cannabis have become more ubiquitous, which can trigger cravings and undermine people’s attempts to reduce cannabis consumption.^15 16^ Cannabis cue-induced craving has been posited to be driven by neuroadaptations within the brain addiction neurocircuitry.^17^

Various functional MRI (fMRI) studies using cue-induced craving tasks demonstrate that individuals who use cannabis (ie, henceforth ‘cannabis users’) compared with controls show increased activity in the addiction neurocircuitry, particularly within a region involved in emotion/craving regulation and decision making, the anterior cingulate cortex (ACC).^18^ Further, there is consistent evidence that cannabis users demonstrate increased subjective craving levels *pre-to-post* cannabis cue-induced craving, and there is emerging evidence that subjective craving levels correlate with brain changes.^18^ Despite the evidence supporting craving-related brain functional alterations in CUD, the extent to which (1) craving-related brain dysfunction may be mitigated and (2) increased activity corresponds to subjective craving in real time remains unclear. Therefore, there is limited understanding of how the craving neurocircuitry is relevant to individual treatment targets, leaving a knowledge gap between a mechanistic understanding of craving and treatment. The present feasibility study aims to use a novel non-invasive neuromodulation tool, real-time fMRI-neurofeedback (fMRI-neurofeedback), that can provide causal, although indirect, evidence on the neurobiological underpinnings of craving in cannabis users.^19^

By providing participants with real-time information on brain function via a brain-computer interface,^20^ fMRI-neurofeedback has been shown to enable participants who use substances to learn to regulate the function of brain pathways relevant to cue-induced craving.^21^ Indeed, alcohol, tobacco and cocaine-using cohorts demonstrate changes *pre-to-post fMRI-neurofeedback* and *between fMRI-neurofeedback conditions* in brain circuitry related to substance cue-induced craving, most consistently in the ACC, but also additional regions (eg, insula, prefrontal cortex (PFC) and striatum). There is also consistent evidence that subjective craving decreases *pre-to-post neurofeedback* and emerging evidence that changes in subjective craving correlate with changes in brain activity, including in ACC.^21^ Therefore, fMRI-neurofeedback holds promise as a tool to modulate the activity of craving-related brain pathways in CUD.

However, as the neurocircuitry of craving may be specific to the psychopharmacological signature of distinct substances,^22^ the feasibility of fMRI-neurofeedback to change craving-related brain function in CUD remains unclear. Second, it remains untested if increasing the activity of craving-related neurocircuitry corresponds to the subjective experience of craving as postulated by neuroscientific theories of addiction.^17^ Third, all studies to date have used high magnetic field strength MRI scanners (3T) fMRI-neurofeedback. Thus, it remains untested if fMRI-neurofeedback using an ultra-high magnetic field strength scanner (7T) can be used to reduce craving-related brain hyperactivity in CUD (or any other substance use disorders).

### Aims

The primary aim of this study is to examine for the first time the feasibility of fMRI-neurofeedback to change brain activity in craving-related pathways (eg, ACC), during the upregulation and downregulation of craving. The secondary aims are to investigate the effect of fMRI-neurofeedback on subjective cue-induced craving in individuals with CUD and the relationship between the observed changes in brain activity and subjective craving.

### Outcome measures

The primary outcome measure is the change in ACC activity during regulation compared with neutral blocks, which will be assessed in real-time and offline. The secondary outcome measures are whole-brain activity during regulation compared with neutral blocks and subjective craving *pre-to-post* neurofeedback runs. The tertiary outcome measure is the association between any observed changes in brain activity and craving.

### Summary

In sum, with the substantial relapse rates of CUD, high prevalence and the increased availability of cannabis products, there is a need to understand the neurobiological mechanisms of craving and develop interventions to target brain hyperactivity during cue-induced craving in CUD. In this regard, fMRI-neurofeedback holds promise to probe the causal neural mechanisms of craving in real-time and to reduce craving-related brain dysfunction.

## Methods

### Study setting

The study will be coordinated by the Neuroscience of Addiction and Mental Health Programme, Healthy Brain and Mind Research Centre, Faculty of Health, Australian Catholic University (ACU). Face-to-face testing will be conducted at the Melbourne Brain Centre Imaging Unit (MBCIU), at the Kenneth Myer Building facility in Parkville, at The University of Melbourne.

### Study design

A within-subject feasibility study will be conducted where 10 participants with CUD, during a single session, receive two interspersed experimental fMRI-neurofeedback conditions to either upregulate or downregulate ACC activity. Individualised feedback targets within the ACC will be identified during a cue-reactivity task prior to fMRI-neurofeedback. A detailed characterisation of mental health, substance use, full-scale IQ (FSIQ) estimate and state changes in craving, anxiety and focus will also be conducted for each participant. This protocol has been developed in line with the CRED-nf checklist (Consensus on the Reporting and Experimental Design of Clinical and Cognitive-Behavioural Neurofeedback Studies)^23^ (see online supplemental information 1). The study will be deemed feasible if the target sample (n=10) is recruited, screened and undergoes the entire testing protocol.

### Eligibility criteria

The target sample will include community members who endorse a moderate-to-severe CUD and who report an attempt to cut down or quit using cannabis in the past 2 years. The following inclusion and exclusion criteria will be applied to the entire cohort.

Inclusion criteria:

- Aged 18 to 55 years.
- Normal-to-corrected vision.
- Fluent in English.
- Willing and able to attend an in-person testing session at MBCIU.
- Willing to abstain from all substances (other than nicotine) for >12 hours prior to testing.
- Daily/almost daily cannabis use for >12 months prior to testing.
- Meeting diagnostic criteria for moderate-to-severe CUD, defined as ≥4 symptoms of CUD in the Structured Clinical Interview for Diagnostic and Statistical Manual of Mental Disorders-5 (DSM-5) Research Version (SCID-5-RV).^24^
- Reported attempt to reduce or quit cannabis use in the past 2 years.

Exclusion criteria:

- Diagnosis of major psychiatric disorders other than severe anxiety and depression due to high co-occurrence in CUD,^25^ as assessed via the Mini-International Neuropsychiatric Interview (MINI).^26^
- Currently prescribed medication that affects the central nervous system (eg, antipsychotics), except for some antidepressants and anxiolytics, due to high co-occurrence in CUD.^25^
- History of a neurological disorder or significant medical condition (eg, epilepsy, stroke, multiple sclerosis, migraine, neurodegenerative disorder, brain tumour).
- History of acquired brain injury or loss of consciousness ≥5 min.
- Any substance use (other than nicotine) in the 12 hours before testing, confirmed via self-report.
- Any substance use (except for cannabis, alcohol and nicotine) in the last 30 days before testing.
- Any significant substance use (other than cannabis, alcohol and nicotine) defined as >50 lifetime episodes of use and/or weekly use over a 3-month period.
- Dependence on a substance other than cannabis and nicotine (eg, Alcohol Use Disorder Identification Test (AUDIT) score ≥19^27^).
- MRI contraindications (eg, surgical clips, pacemaker).
- Currently breastfeeding or pregnant.
- FSIQ estimate score <80, as estimated via the Wechsler Test of Adult Reading (WTAR).^28^

### Sample size

The target sample of 10 participants with moderate-to-severe CUD is due to the pragmatics of testing the feasibility of fMRI-neurofeedback. The target sample equates to recommended sample sizes for pilot studies.^29^ The results will be used to inform power analyses and sample size estimations for larger-scale studies.^30 31^

### Recruitment and testing dates

The approximate start date for the trial will be May 2025, data collection is expected to conclude in October 2025, with an estimated trial duration of 6 months. Study advertisements will occur via printed and online flyers distributed by student researchers in the general community and university campuses of the Melbourne metropolitan area, Australia and via public platforms (eg, Google Ads, Gumtree, Facebook, university websites and others). The study flyers will direct all people interested in participating in the study to an online screening survey outlined below.

### High-level description of the testing protocol

**Figure 1:**
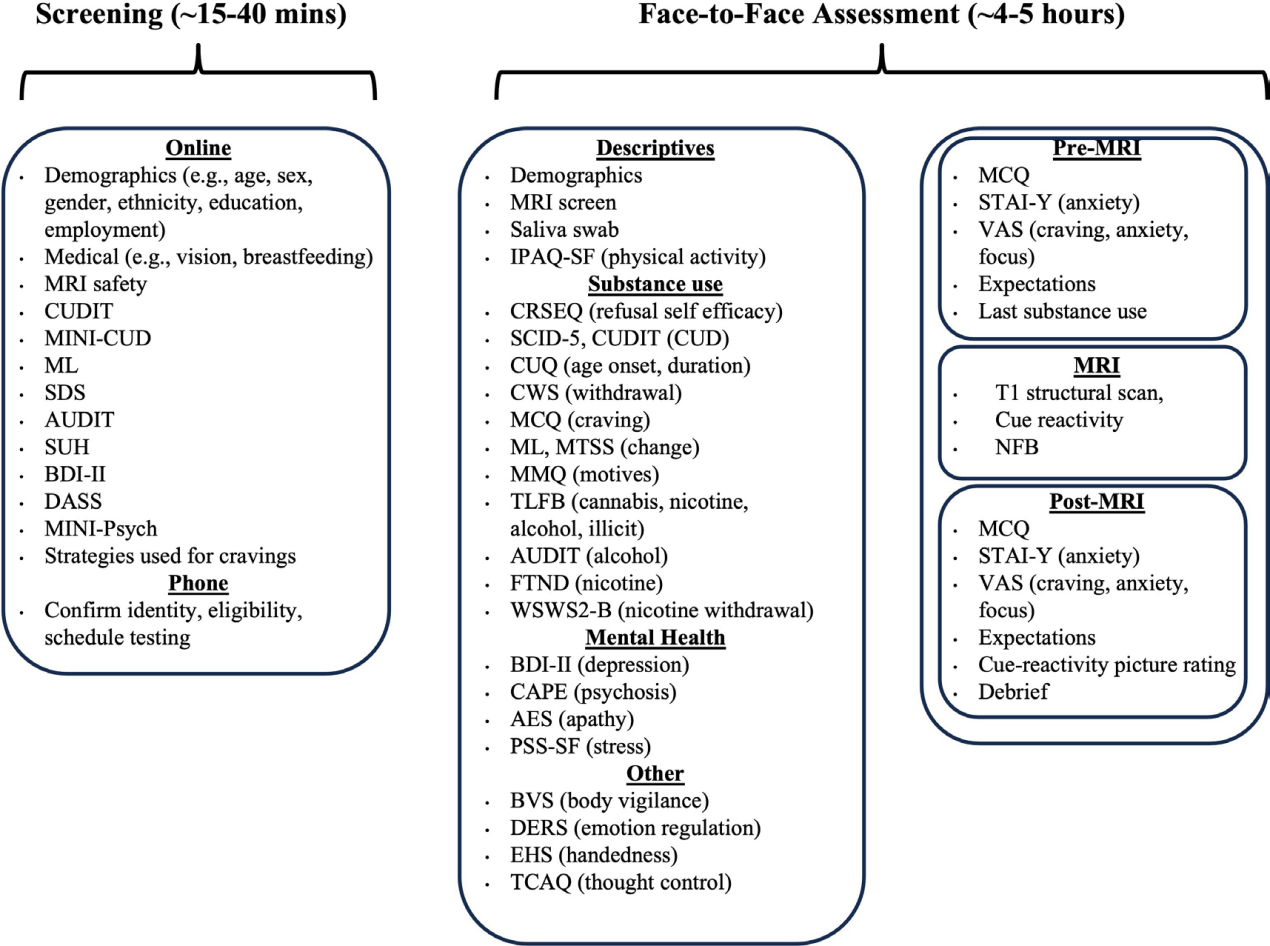
Overview of the assessment protocol and included measures. AES, Apathy Evaluation Scale^50^; AUDIT, Alcohol Use Disorders Identification Test^27^; BDI-II, Beck Depression-II^68^; BVS, Body Vigilance Scale^52^; CAPE, Community Assessment of Psychotic Experiences^51^; CRSEQ, Cannabis Refusal Self-Efficacy Questionnaire^47^; CUDIT, Cannabis Use Disorder Identification Test^33^; CUQ, Cannabis Use Questionnaire^69^; CWS, Cannabis Withdrawal Scale^45^; DASS, Depression, Anxiety and Stress Scale^36^; DERS, Difficulties in Emotion Regulation Scale^55^; EHS, Edinburgh Handedness Inventory - Short Form^70^; FTND, Fagerström Test for Nicotine Dependence^43^; IPAQ-SF, International Physical Activity Questionnaire Short form^53^; MCQ, Marijuana Craving Questionnaire^39^; MINI, Mini-International Neuropsychiatric Interview^26^; ML, Marijuana Ladder^35^; MMQ, Marijuana Motives Questionnaire^46^; MTSS, Motivation to Stop Scale^44^; NFB, neurofeedback; PSS-SF, Perceived Stress Scale-short form^49^; SCID-5, Structured Clinical Interview of DSM-5-research version^71^; SDS, Severity of Dependence Scale (Cannabis)^72^; STAI-Y, State-Trait Anxiety Index-Y Form^38^; SUH, Substance Use History; TCAQ, Thought Control Ability Questionnaire^54^; TLFB, Timeline Follow Back^42^; VAS, Visual Analogue Scale; WSWS2-B, Wisconsin Smoking Withdrawal Scale 2-Brief Version.^48^

As shown in figure 1, participants will undergo a thorough selection for study inclusion using online and phone screening. Upon study inclusion, the face-to-face testing protocol will comprise three main parts:

1. ∼1–1.5 hour assessment session which must be administered before the experiment takes place (eg, participant information letter (PIL), informed consent, behavioural practice of the task).
2. ∼1 hour fMRI scanning session which includes a structural scan, cue-reactivity task and fMRI-neurofeedback training (two runs of upregulation and two runs of downregulation). Short questionnaires will be administered immediately before, during and after the scan.
3. ~2–3-hour assessment session which can be administered either before or after the fMRI-neurofeedback training, containing the measures required for sample characterisation (eg, substance use, mental health, and cognition).

### Screening

Interested members of the community will be directed to a ∼15–40 min online screening survey to determine their eligibility against the study-specific inclusion and exclusion criteria. The measures included in the online screening are overviewed in figure 1 and described in detail below. Several metrics were administered at different stages of the testing protocol to serve distinct purposes: during online screening to ascertain study eligibility, and during face-to-face testing to measure key variables relevant for describing the sample and interpreting the data (eg, substance use and related problems as assessed via the Cannabis Use Disorder Identification Test (CUDIT) and AUDIT).

### Online screening measures

#### Sociodemographic and health

Demographic data (eg, age, date of birth, English/other languages spoken, prescription glasses, prescription medication, including medicinal cannabis and other psychoactive medication).

- Pregnancy/breastfeeding status (yes/no).
- Lifetime prescription medication (yes/no, type and details).
- Lifetime personal diagnoses of psychosis, depression or any other psychological disorder that requires ongoing medication or treatment (yes/no).
- Lifetime diagnoses of psychiatric disorders in parents or siblings (yes/no).
- Previously seen psychologist/psychiatrist/counsellor or undergone related therapy type (yes/no).
- MRI safety Screening Questionnaire (provided by MBCIU) and information regarding the MRI scanning process including incidental findings and participant general practitioner details.
- Previous study participation (yes/no and details).

#### Substance use and related problems

- Past cannabis use details, for example, most frequent use of cannabis in the past 3 months, 12 months, and life.
- Levels of alcohol consumption, drinking behaviour, and alcohol-related problems will be assessed using the AUDIT.^27^
- The Substance Use History questionnaire will be used to assess the frequency and dosage of psychoactive substance use over the lifetime, past 12 months, and past 3 months. The tool has been adapted from the Drug History Questionnaire.^32^
- The presence and severity of CUD will be assessed using the MINI V.7.0.2 Substance Dependence/Abuse (Cannabis) Subscale.^26^ The measure includes 12 items relating to cannabis use, where CUD severity is classified through the number of endorsed criteria (1–3=mild, 4–5=moderate, 6≥ = severe).
- The CUDIT-Revised (CUDIT-R) is an 8-item cannabis use assessment tool that will be used to further assess CUD severity.^33^
- Previous cannabis quit attempts in the past 12–24 months (yes/no).
- The Severity Dependence Scale is a 5-item tool that will be used to assess the extent of thoughts, worries and difficulties controlling cannabis use over the previous 3 months.^34^
- The Marijuana Ladder (ML) is a single-item measure that will be used to assess participants’ motivation to stop using cannabis (ie, their readiness to change).^35^

#### Mental health

- The MINI V.6.0.0 Screen is a 24-item measure that will be used to screen for 17 of the most common psychiatric DSM-IV disorders (eg, depression, psychosis) and includes items on suicidal ideation.^26^ It should be noted that participants who endorse MINI items 3 and 4 (relating to suicidality) will be screened out.
- The Depression, Anxiety and Stress Scale is a 21-item measure that will be used to assess depression, anxiety and stress.^36^

#### Phone screening and scheduling testing

Potentially eligible participants will be identified through the online screening described above. Any queries concerning participant eligibility will be resolved via discussion with the study chief investigator (CI) and research team. Next, the student researchers will call prospective participants to confirm eligibility, map methods/techniques used by the participant to increase and decrease cannabis cravings and schedule an assessment time. During this call, participants will also be described MBCIU’s incidental findings process. In brief, the research team (ie, the principal investigator (PI)) is required to inform participants should an incidental finding be discovered during MRI scanning. The PI will be able to prepare a letter for the participant to take to their general practitioner, should they wish to discuss this further.

Testing will be scheduled at a mutually convenient time and date. A follow-up email will be sent to participants, with instructions on how to get from the participant’s location to the testing site in Google Maps and a short video of the fMRI-neurofeedback process so that participants can familiarise themselves with the task. Testers will manage testing times via a shared Google calendar. Message Media-Hub (https://hub.messagemedia.com) will be used to schedule SMS reminders to participants the day before testing with the information on testing location and time.

### Face-to-face assessment

#### Overview

Face-to-face testing sessions will be conducted by trained student researchers and are expected to last ∼4–5 hours. Participants will complete an assessment session that includes fMRI-neurofeedback as well as a battery of validated questionnaires and semistructured interviews to profile substance use, mental health, FSIQ estimate, techniques to increase and decrease craving, and intervention-related tasks and questionnaires.

**Figure 2:**
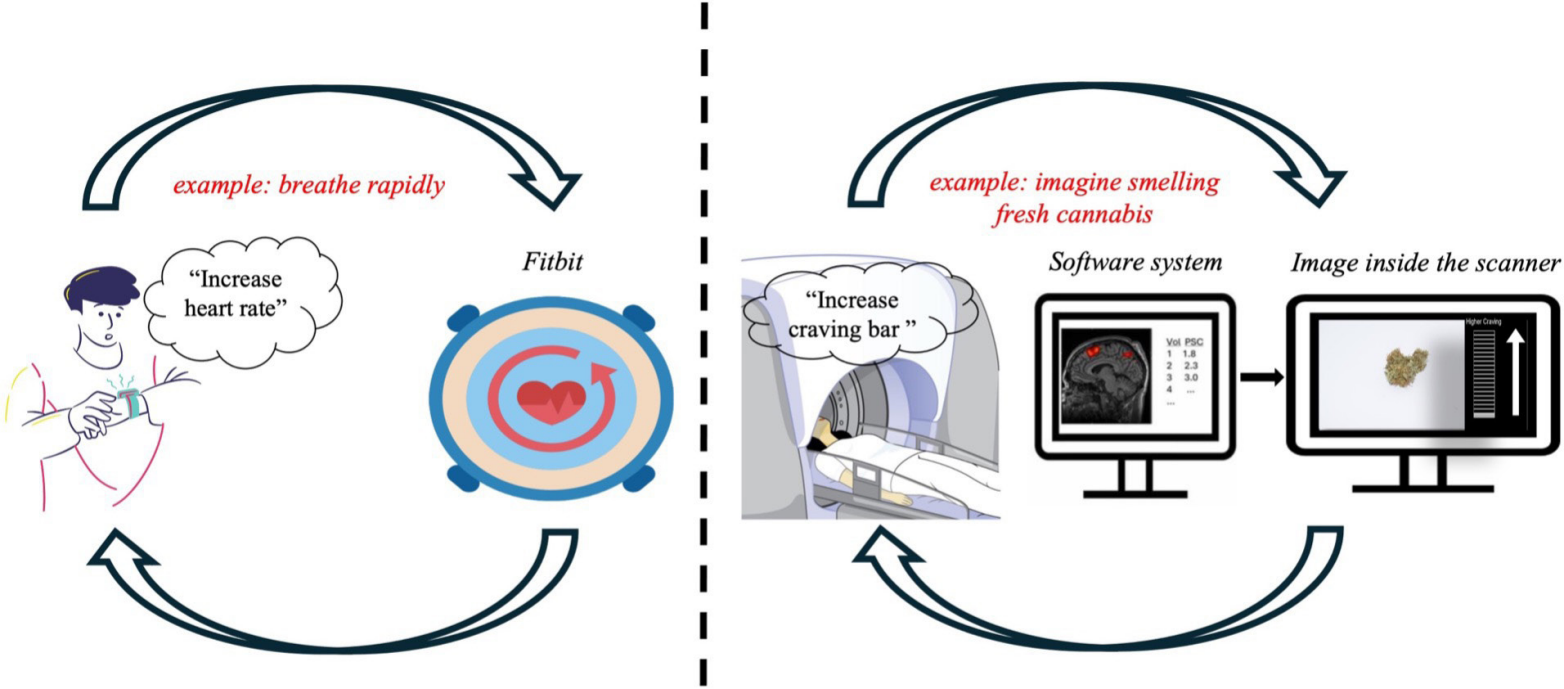
Schematic explaining the fMRI-neurofeedback process using the analogy of a heart rate monitor. Participants will be told to imagine that they are wearing a smartwatch depicting their heart rate. Someone comes along and asks them to increase their heart rate. They will be asked to think of some things they could do to achieve this and to use the information displayed on their smartwatch to tell them how well they are doing. Participants will be told that fMRI-neurofeedback works in the same way. They will be asked to imagine that they are in a scanner, and they can see a craving bar that represents their brain activity linked with craving. They will be asked to think of some ways that they could increase or decrease the craving bar and to use the craving bar to tell them how well they are doing. fMRI, functional MRI.

The following procedure will be applied before participants enter the MRI scanner. First, the tester will ask the participant to review and clarify essential study details explained in the PIL and provide written informed consent to participate in the study. Second, the tester will ask the participant to complete a saliva swab (*Andatech-DS08*) to indicate the presence or absence of THC metabolites. The swab will also confirm the presence or absence of 6 other illicit substances (ie, amphetamine, cocaine, synthetic THC, benzodiazepines, methamphetamine, opiates) and alcohol. Researchers will explain the MRI processes, including the structural scan and cue-reactivity fMRI task. Researchers will outline the neurofeedback process, using an analogy of biofeedback-mediated regulation of heart rate (see figure 2). To prepare participants for the fMRI-neurofeedback process, they will be shown two videos of the task to be performed. The first video will contain audio instructions for task performance. Participants will be informed that the observed changes in the craving bar are shown as an example and are not their actual brain activity, which will only be recorded when they are inside the scanner. The second video will not contain audio instructions or a craving bar, and participants will have the opportunity to practise the task and perform the craving regulation methods/techniques (one of the videos can be found in the online supplemental information). Participants will be provided with the verbatim instructions provided in online supplemental information 1 (Section 1.1) for the behavioural practice. Before and after this ‘behavioural practice’, participants will be asked several questions relating to their current state of craving, focus, anxiety, task comprehension and confidence, and methods/techniques used to increase and decrease craving. After changing into scrubs, participants will be provided with the verbatim instructions provided in online supplemental information 1 (Section 2.2).

**Figure 3:**
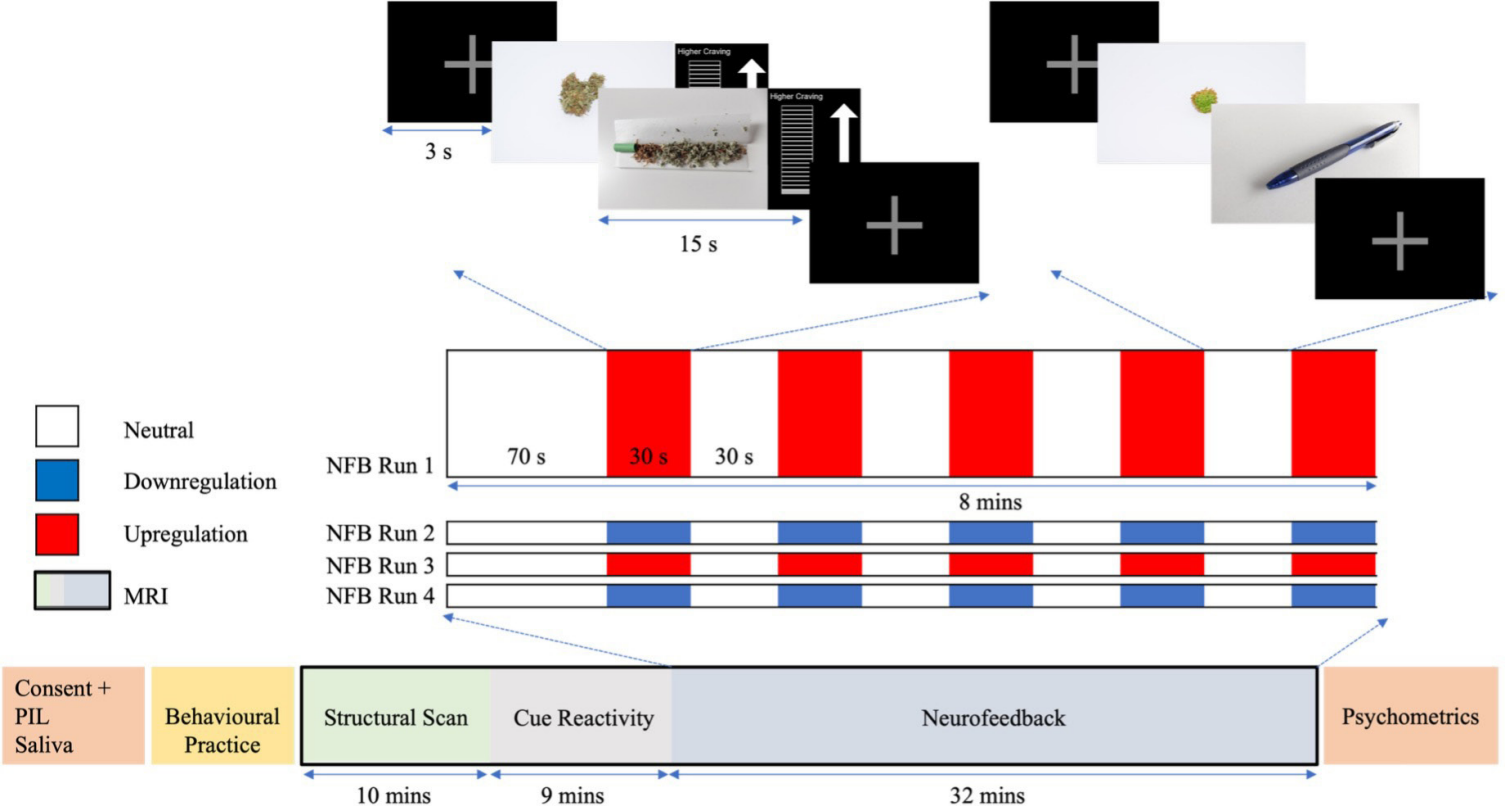
Overview of neurofeedback experimental design. The MRI scanning session included a structural scan, a cue-reactivity fMRI task to localise which part of the region-of-interest the participant activated the most while viewing cannabis vs. neutral images, and a fMRI-neurofeedback session. Assessment of several psychosocial variables, were conducted before, during and after scanning. Saliva testing for the presence of THC was conducted at the start of testing. The fMRI-neurofeedback session will consist of two runs of upregulation of brain activity, and two runs of downregulation of brain activity in the region-of-interest, with each run lasting approximately 8 minutes. Each fMRI-neurofeedback run will contain alternating blocks of regulation and non-regulation, during which either two cannabis-related or two neutral images will be displayed for 15 seconds each. NFB, fMRI-neurofeedback; PIL, participant information letter.

Next, participants will undergo the MRI scanning session that includes fMRI-neurofeedback. The MRI scan will measure brain structure and function and will be used to deliver the fMRI-neurofeedback experiment. As illustrated in figure 3, each scanning session will consist of a T1-structural scan, a cue-reactivity task and an fMRI-neurofeedback session. The cue-reactivity scan will last for roughly 9 min, during which participants will be asked to passively view 30 cannabis-related and 30 non-cannabis-related images.^37^ The cue-reactivity scan will be used to functionally localise the top-activated voxels within the region of interest (ROI) for each participant (described in more detail below). The fMRI-neurofeedback session will last approximately 32 min and comprises four runs (two upregulation and two downregulation; 8 min and 3 s each). Each run will contain a block design that will consist of a baseline first block of 70 s, followed by 5 blocks of non-regulation (∼30 s each) and 5 of regulation (∼30 s each), with each block being interspersed with a 3 s fixation cross. During regulation blocks, participants will view cannabis-related images and be asked to either increase (upregulate) or decrease (downregulate) a craving bar that is continuously presented and represents ROI activity. During non-regulation (‘neutral’) blocks, participants will be asked to simply watch non-cannabis-related (‘neutral’) images, and no craving bar will be presented. In both regulation and neutral blocks, two images will be presented for 15 s each. Before the first neurofeedback run and after each subsequent run, participants will use a button box to rate their levels of craving, focus and anxiety via Visual Analogue Scales (VAS) that will appear on a screen. Before and after the neurofeedback session, participants will be asked to complete several questions, relating to state changes in anxiety, craving and expectations/perceptions of the training.

Following the conclusion of the MRI scanning, participants will complete questionnaires and semistructured interviews for sample characterisation. This includes an FSIQ estimate assessment, semistructured interviews and self-report questionnaires relating to substance use, mental health, and cognition.

#### Pre-to-post MRI and behavioural practice measures

Several short questionnaires were administered to participants immediately before and after the behavioural neurofeedback practice and the MRI scan to map changes in subjective psychological states, state anxiety and cannabis craving, while also assessing their expectations and perceptions of the fMRI-neurofeedback training.

- The State-Trait Anxiety Index-Y Form (STAI-Y) is a 20-item measure that will be used to assess state anxiety.^38^ The STAI-Y will only be administered before and after the scan (ie, not before and after the behavioural practice).
- The Marijuana Craving Questionnaire-Short Form is a 12-item measure that will be used to assess the magnitude of current cannabis craving.^39^

The following VAS items will be used to assess state cannabis craving, anxiety, and focus. VAS items relating to anxiety and focus have been included as they are key confounds of craving and neurofeedback learning, respectively.^40 41^

- Cannabis craving (ie, How much do you feel like smoking cannabis right now? From 1=Not at all; 10=Extremely).
- Anxiety (ie, How anxious do you feel right now? From 1=Not at all; 10=Extremely).
- Focus (ie, How focused are you right now? From 1=Not at all; 10=Extremely).

For both increase/upregulation and decrease/downregulation conditions, several items will be used to assess participants’ expectations and perceptions of the behavioural practice and fMRI-neurofeedback training, including participants’ understanding of task instructions and instructions on using craving techniques, the methods/techniques participants think will be effective in increasing/decreasing craving and the craving bar, the degree to which the level of the craving bar reflects subjective craving, and the likelihood of recommending fMRI-neurofeedback to a friend who is looking to manage feelings of craving/desire to use cannabis.

#### Scanning measures

The previously described VAS items on craving, anxiety and focus will be administered to participants during the scanning, at the beginning and end of the cue-reactivity, and after each neurofeedback run.

#### Baseline face-to-face assessment measures

Following the MRI scan and pre-to-post assessments, participants will undergo the following sample characterisation measures.

#### Sociodemographic, medical and handedness data

Questionnaires will be administered to measure sociodemographic variables, including age in years, sex, gender (ie, male, female, non-binary or other), ethnicity, and the number of education years.

#### Substance use and related problems

The following semistructured interviews will be administered for sample characterisation.

- The SCID-5-RV^24^ is an 11-item semistructured interview that will be used to measure CUD according to DSM-5 criteria. This will be used to confirm a diagnosis of moderate-to-severe CUD as well as the total number of CUD symptoms.
- The Timeline Follow Back will be used retrospectively, with the aid of a calendar, to estimate the number of days of substance use and quantity of any psychoactive substance used over the past 30 days, as well as hours since last use.^42^

The following questionnaires will be administered to measure substance use and related problems.

- The AUDIT will be used to measure levels of alcohol consumption, drinking behaviour and alcohol-related problems in the past 12 months.^27^
- The Fagerström Test for Nicotine Dependence is a 6-item tool that will be used to assess nicotine use and nicotine dependence, where a score of ≥3 indicates the presence of nicotine dependence.^43^
- The CUDIT-R is an 8-item tool that will be used to assess the level of CUD severity level at the time of the face-to-face assessment session (past 6 months).^33^
- The Motivation to Stop Scale will be used to assess the desire and/or intention to stop using substances.^44^
- The Cannabis Withdrawal Scale is a 19-item tool that will be administered to assess participants’ levels of cannabis withdrawal over the past 24 hours.^45^
- The Marijuana Motives Questionnaire is a 25-item tool that will be used to measure the motivations to use cannabis and potentially related consequences.^46^
- The Cannabis Refusal Self-Efficacy Questionnaire is a 14-item measure where participants rate their ability to handle situations where they might want to use cannabis (eg, when at a party, when friends are smoking^47^).
- The ML will be used to assess participants’ motivation to stop using cannabis (ie, their readiness to change).^35^
- The Wisconsin Smoking Withdrawal Scale 2-Brief Version is a 6-item measure that will be used to assess participants’ level of nicotine withdrawal in the past 24 hours.^48^

#### Mental health and well-being

- The Perceived Stress Scale is a 4-item tool that will be used to measure participants’ perceptions of stress in the past month.^49^
- The Apathy Evaluation Scale is an 18-item measure that will be used to measure general feelings of apathy.^50^
- The Community Assessment of Psychic Experiences is a 42-item measure that will be used to measure the rate and distress of positive and negative psychotic symptoms.^51^
- The Beck Depression-II will be used to measure participants’ experiences of depression in the past 2 weeks.^50^

#### Other

- The Body Vigilance Scale is a 4-item measure used to assess attention to multiple internal bodily sensations over the past week, including the degree of focus and average time spent attending to these sensations.^52^
- The International Physical Activity Questionnaire-short form is a 7-item measure that will be used to measure the frequency and duration of vigorous activity, moderate activity, walking and sitting over the previous 7 days.^53^
- The Thought Control Ability Questionnaire is a 25-item tool that will be used to measure perceived control over thoughts.^54^
- The Difficulties in Emotion Regulation Scale-16-item version questionnaire will be used to assess emotion regulation.^55^
- The WTAR is a standardised measure that will be used to estimate premorbid IQ.^28^ It contains 50 words printed in order of increasing difficulty, and participants are asked to read each word aloud; the test is discontinued after 12 consecutive incorrect pronunciations.

#### Debrief and reimbursement

Upon completion of the assessment, study researchers will debrief with the participant and participants will be reimbursed with a Coles/Myer voucher valued at $A150 and provided with a three-dimensional print of their brain. During the debrief, participants will be provided with the contact information of the CI should they wish to further discuss the project, the Manager of the Human Research Ethics Committee (HREC) and the Office of the Deputy Vice-Chancellor (Research) at ACU should they have any issues or concerns about the conduct of the project. They will also be provided with support services, such as Beyond Blue and Headspace, should they require mental health support.

### Neuroimaging metrics

All structural and functional brain data will be measured using MRI. Participants will be scanned on a 7 Tesla MRI scanner (Siemens Magnetom 7T plus) at the MBCIU using an 8-channel transmit, 32 channel receive head coil.

#### T1-weighted structural MRI protocol

Brain structure will be measured to spatially register the ROI for fMRI-neurofeedback at the individual level. A 7 min T1-weighted scan will be acquired using a 3D-MP2RAGE sequence (0.75 mm × 0.75 mm × 0.75 mm; echo time (TE)/repetition time (TR)=2 ms/5000 ms).^56^ After the structural scan, participants will be shown the example neurofeedback video that was presented during behavioural practice, while 10 TRs of bidirectional (Anterior (A)–Posterior (P)) phase-encoded spin-echo images and reverse phase-encoded fMRI images (A–P) will be collected (5 min) for distortion correction purposes in the analysis pipeline.

#### Functional MRI protocol

Brain activity during cue-reactivity and fMRI-neurofeedback will be acquired using a multiband gradient-echo EPI sequence (1.6 mm isotropic; TE/TR=22 ms/1000 ms; multiband acceleration=6; field-of-view = 208 mm; matrix size=130 × 130; 84 slices; slice thickness=1.6 mm; flip angle=45^0^; P–A phase encoded).^57^ Concurrent to fMRI acquisition, participants’ respiratory signals will be recorded using a Siemens MRI-compatible respiration belt worn around the abdomen. Similarly, cardiac measurements will be recorded using a Siemens MRI-compatible pulse oximetry sensor worn on a fingertip.

#### Real-time functional localisation during cue-reactivity

The real-time fMRI preprocessing and analysis for functional localisation and fMRI-neurofeedback will be conducted using the software TurboBrain Voyager (TBV) V.4.2. The timed visual display of cues, instructions and feedback scores inside the scanner will be presented using the MATLAB Psychtoolbox software (V.3.1). Preprocessing in TBV includes the coregistration to anatomical and Montreal Neurological Institute space, standard 6-parameter motion correction, spatial smoothing, linear detrending and physiological control through nuisance regression using activity from a confound ROI (described in further detail).

As overviewed in figure 3, an event-related cue-reactivity fMRI task (∼9 min) will be used to map the most highly activated part of the ROI (ACC) for each participant in real-time. Participants will be asked to view 30 cannabis-related and 30 non-cannabis-related images that have been previously validated by the wider research team.^37^ Cannabis-related images contain visuals of cannabis-related paraphernalia or smoking behaviours, while non-cannabis-related images contain neutral cues, including cooking utensils or stationary items. All cannabis and non-cannabis images will be matched in terms of complexity, size, brightness, luminance and activity type. The ACC will initially be defined based on the functionally significant voxels (cannabis picture>neutral picture) within the ACC (Harvard-Oxford Atlas) according to the data from the team’s previously collected cue-reactivity study in individuals with CUD.^37^ The highly activated voxels (t-threshold >2) within this initial ACC ROI will be determined in TBV following the cue-reactivity scan (t-contrast cannabis picture>neutral picture). These highly activated voxels (henceforth, ACC) will act as the individualised ROI, the activity of which will be continuously displayed to the participant during fMRI-neurofeedback training.

#### Real-time fMRI-neurofeedback

As described in detail above, the neurofeedback training will consist of four runs (two upregulation and two downregulation). To reduce the risk that participants complete the neurofeedback training with amplified levels of craving, each training session will end with a downregulation run. Participants will be asked to increase or decrease a craving bar that is continuously presented with cannabis-related images and represents ACC activity. The blocks of neurofeedback regulation will be interspersed with non-regulation blocks where neutral images are presented (‘neutral blocks’). In a similar fashion to the cue-reactivity task, all cannabis and non-cannabis images will be visually matched.

The fMRI-neurofeedback setup of this study has previously been used to successfully investigate fMRI-neurofeedback to optimise self-guided meditation practice in novice meditators.^58^ In brief, the setup involves three principal systems: the MRI console, the software system that processes the fMRI data in real-time (ie, TBV) and a stimulus PC. Transmission Control Protocol communication will be used to enable real-time communication between these three systems. Inside the scanner, 7T MRI Digital Imaging and Communications in Medicine (DICOM) images will be collected from participants while they view substance-related stimuli. These raw DICOM images are passed to an external server, and then onto TBV for processing. The processed outputs from TBV are saved in a dedicated feedback folder which can be accessed using MATLAB Psychtoolbox on the stimulus PC. The stimulus PC can then present the latest TBV outputs to participants in the form of visual feedback (eg, craving bar).

The real-time percentage signal change (PSC) of the target ACC ROI will be calculated in TBV using an incremental general linear model (GLM) that includes six motion regressors after detrending, with respect to a baseline 70 s block during which one neutral image will be presented. The PSC will be estimated from the top 33% of activated voxels within the ACC during regulation compared with neutral blocks. The PSC (A) will be calculated using the following ‘sliding window’ equation:


$$A=0.5\;\left(V_{i}\right)+0.25\;\left(V_{i-1}\right)+0.125\left(V_{i-2}+V_{i-3}\right)$$

where, V_i_=PSC from current volume; V_i−1_=PSC from previous volume; V_i−2_=PSC from second last; V_i−3_=PSC from third last.

A confound ROI will be used for real-time physiological correction. The confound ROI builds on a midline confound region previously tested with fMRI neurofeedback^58^ but only includes regions within a cerebrospinal fluid mask derived from the Wake Forest University PickAtlas (ie, excludes grey matter regions^59^). The PSC from the confound ROI will be calculated in TBV and used as the predictor of a cumulative GLM in MATLAB where the ACC PSC is a response variable. The resultant residual ACC PSC will be continuously presented to participants on a craving bar with 20 levels.

## Ethics and dissemination

### Research ethics approval

The project was run in line with the *National Statement on Ethical Conduct in Human Research* (2007), the *International Council for Harmonisation Guideline for Good Clinical Practice(ICH GCP),* and the principles that have their origin in the Declaration of Helsinki. Ethics approval was obtained in April 2025 by the HREC of the ACU, Melbourne, Australia (ID:2023–3338).

### Patient and public involvement

Individuals with lived experience of cannabis use were consulted at various stages throughout protocol development and study design. The research team met with several ‘lived experience’ participants who provided their perspectives on multiple aspects of the study, including recruitment (eg, advertisement flyers), methods/techniques that could be used to increase and decrease craving for cannabis, the language used in the protocol (eg, participants suggested replacing the term ‘craving thermometer’ with ‘craving bar’), reimbursement and potential barriers to participation (eg, participants suggested the option to conduct weekend assessments to increase the likelihood of participation of individuals who are employed full-time). All lived experience participants were reimbursed with Coles Myer vouchers valued at $A50 for their time and input.

### Informed consent

Participants will be informed about the study components over the phone to ensure that they will be familiar and comfortable with all aspects of the study and to provide the opportunity for participants to ask any questions they might have. They will be informed that they can withdraw at any time with no consequences to the relationship with the study investigators. They will then receive a copy of the study PIL via email. At the start of each face-to-face testing session, study researchers will review the PIL with the participants before proceeding to obtain written informed consent.

### Participant fatigue and emotional strain

An assessment session of between 4 and 5 hours could be fatiguing for participants, especially those who frequently use substances and who may arrive with elevated baseline anxiety.^60 61^ To reduce the effects of fatigue, all participants will be given the opportunity to have breaks at various points before and after MRI scanning and will be provided with light refreshments. Similarly, several systems need to be in place should participants experience discomfort with any part of testing, including but not limited to craving and anxiety: researchers will offer to pause or cease the session and remind participants they can leave voluntarily without any consequences for their relationship with the team. Testers will be trained in techniques to support participants in these instances, such as compassionate communication, brief grounding techniques (eg, grounding/progressive muscle relaxation), and mental health aid. A trained clinician will be made available for every testing session to support the tester in managing participants’ anxiety/craving. Trained testers will also inform participants of free services with 24/7 availability at the testing location (eg, Lifeline and Beyond Blue), the details of which will be given in the PIL and debrief forms.

### Study governance

The study will be monitored through an overarching multidisciplinary senior research team that meets on a weekly to fortnightly basis (VL, CS, GP). Additional input will be received from international investigators on the trial component of the study on a need basis (SKK, AZ, BAM, SG). A research team group comprising student researchers (and support staff on a need basis) will meet weekly with the PI (VL) to discuss the day-to-day operations and logistics of the project. EM will analyse the data from the downregulation condition, while AHD will analyse the data from the upregulation condition.

### Data access

Selected members of the study research team will have access to the collected data as required. The neuroimaging data will be sent to the analysis servers (ACU) in de-identified form using DARIS (https://dataservices.research.unimelb.edu.au/services/41/). Then, for the neuroimaging data, a secure copy (DARIS) is retained by the MBCIU for management of rare incidental findings in accordance with HREC approved protocol. For neuroimaging data processing, data will be stored and processed in a multi-modal Australian ScienceS Imaging and Visualization Environment (MASSIVE) high performance computing infrastructure.^62^ For further processing, neuroimaging data from this research project will be stored by the researchers on an ACU secure server; as well as other data types (eg, information on saliva sample results, behavioral and cognitive data). All electronic data will be securely stored with restricted access at ACU. Hard copy of the data will be retained at the ACU Melbourne Campus.

To maintain confidentiality, data from this project with identifying information — consent and reimbursement forms — will be stored separately in locked filing cabinets accessible by the study Chief Investigator (VL). All other data collected will be allocated an alphanumerical code (and not participants’ identifying information), so participants’ cannot be personally identified via their data.

### Dissemination policy

The CONSORT (Consolidated Standards of Reporting Trials) guidelines will be followed for reporting the results of this study.^63^ Briefly, the findings will be submitted to peer-reviewed academic journals, while conference presentations, media releases and scientific meetings will also be organised. We also plan to leverage the wider research team’s networks, so that the findings can be shared with individuals with lived experience of cannabis use and CUD as well as caregivers and clinicians.

## Discussion

As a feasibility study, we acknowledge that the described protocol comes with several methodological limitations. First, a relatively small sample size (n=10) will be included. Future research should include more participants to increase the reliability with which small-to-medium effects may be observed.^64^ Similarly, the primary objective of this study is to investigate if fMRI-neurofeedback can change craving-related brain activity in cannabis users. However, the lack of a sham/mock fMRI-neurofeedback control group means that we cannot confirm whether the effects reported here are specific to fMRI-neurofeedback or non-specific neurobehavioural changes related to brain training, or from being in the scanner.^65^ We recommend that future studies include an active-control group to confirm that the observed neural effects are specific to fMRI-neurofeedback and do not relate to more general processes. For instance, a cross-over design in a larger sample, where two intervention conditions (eg, neurofeedback, mock) are administered in a counterbalanced order to two matched participant groups, is warranted. Further, the mock neurofeedback condition should not be related to either visual-cue processing in substance users^18^ or self-regulation processes underlying fMRI-neurofeedback training.^66^ Third, participants will undertake a single fMRI-neurofeedback session, reducing the likelihood that the training will induce lasting neural and behavioural effects.^67^ Fourth, the absence of a transfer run and behavioural testing after the training day limits our ability to investigate the transferability of the neurofeedback-learnt craving regulation techniques to a non-neurofeedback context. Replication studies should integrate a transfer run and behavioural follow-ups to test these notions.

To our knowledge, this is the first protocol for (1) an fMRI-neurofeedback study in cannabis users; and (2) ultra-high magnetic field strength scanner (7T) fMRI-neurofeedback in any substance-using population. The study relies on prominent neuroscientific theories of addiction, emerging evidence on people who use cannabis, and extensive reviews of the relevant evidence conducted by members of the research team. By setting the foundation for future replication studies and clinical trials, the potential implications of this feasibility study are wide-ranging. For instance, the upregulation condition of our neurofeedback training holds promise to provide some much-needed causal evidence to support the proposed neurocircuitry of craving for cannabis. Similarly, the downregulation condition may hold promise as a non-invasive tool to mitigate craving and restore brain function in CUD.

## Supplementary material

10.1136/bmjopen-2025-105854 online supplemental file 1

10.1136/bmjopen-2025-105854 online supplemental file 2
