# Supplementary material for: CannChange: a protocol for a feasibility study using fMRI-based neurofeedback to change the neurobiology of craving in cannabis use disorder
Source: BMJ Open. 2025 Aug 31;15(8):e105854. doi: 10.1136/bmjopen-2025-105854 (PMC12406938; doi:10.1136/bmjopen-2025-105854)
Supplement: online supplemental file 1 [file bmjopen-15-8-s001.docx]

1. CRED-NF Checklist

| **Domain** | **Item #** | **Checklist item** | **Reported on page #** |
| --- | --- | --- | --- |
| **Pre-experiment** | | | |
|  | 1a | Pre-register experimental protocol and planned analyses | ALL |
|  | 1b | Justify sample size | 11 |
| **Control groups** | | | |
|  | 2a | Employ control group(s) or control condition(s) | NA |
|  | 2b | When leveraging experimental designs where a double-blind is possible, use a double-blind | NA |
|  | 2c | Blind those who rate the outcomes, and when possible, the statisticians involved | NA |
|  | 2d | Examine to what extent participants and experimenters remain blinded | NA |
|  | 2e | In clinical efficacy studies, employ a standard-of-care intervention group as a benchmark for improvement | NA |
| **Control measures** | | | |
|  | 3a | Collect data on psychosocial factors | 20 |
|  | 3b | Report whether participants were provided with a strategy | 16 |
|  | 3c | Report the strategies participants used | NA |
|  | 3d | Report methods used for online-data processing and artifact correction | 26 |
|  | 3e | Report condition and group effects for artifacts | NA |
| **Feedback specifications** | | | |
|  | 4a | Report how the online-feature extraction was defined | 26 |
|  | 4b | Report and justify the reinforcement schedule | 26 |
|  | 4c | Report the feedback modality and content | 26 |
|  | 4d | Collect and report all brain activity variable(s) and/or contrasts used for feedback, as displayed to experimental participants | 26 |
|  | 4e | Report the hardware and software used | 23 |
| **Outcome measures** | | | |
| Brain | 5a | Report neurofeedback regulation success based on the feedback signal | NA |
|  | 5b | Plot within-session and between-session regulation blocks of feedback variable(s), as well as pre-to-post resting baselines or contrasts | NA |
|  | 5c | Statistically compare the experimental condition/group to the control condition(s)/group(s) (not only each group to baseline measures) | NA |
| Behaviour | 6a | Include measures of clinical or behavioural significance, defined a priori, and describe whether they were reached | 20 |
|  | 6b | Run correlational analyses between regulation success and behavioural outcomes | NA |
| **Data storage** | | |  |
|  | 7a | Upload all materials, analysis scripts, code, and raw data used for analyses, as well as final values, to an open access data repository, when feasible | NA |

1. Instructions provided to participants
   1. During explanation of neurofeedback process

“We are now going to practice the task that you will do inside the scanner during the brain training. Your task will be to change your brain activity when you see cannabis images. When you will be in the scanner, you will also see a thermometer that will indicate your brain activity.

We are going to show you a video and you can practice strategies to try and increase your craving while watching images of cannabis. By strategies we mean, thoughts that increase your desire to use cannabis. For example, some people find that a) ‘thinking about the positive effects of using cannabis’ or b) ‘imagining being close to cannabis, how it would smell, how it would feel’ increases their craving.

For some other cannabis pictures, we will ask you to decrease your craving. Some people find that thinking of the future/long-term negative effects of using cannabis helps to reduce their craving. Other strategies or thoughts that you could try might include a) thinking of other things (like counting numbers); b) noticing your thoughts of craving and letting them go; c) pushing the thoughts of craving out of your mind; d) focusing on your breathing.

When you see non-cannabis images please just look at the images, no need to try to change your brain activity. This practice will take about 5 minutes”.

- 1. After participant changes into scrubs and before entering the scanner

“We are soon going to start the brain training task, where you will see a thermometer and some images. As we just mentioned, the thermometer represents your brain activity associated with craving. When you see images related to cannabis, try to use strategies that you have found to increase or decrease your craving. But if these don’t change the thermometer, try something else”
