# Supplementary material for: CannChange: a protocol for a feasibility study using fMRI-based neurofeedback to change the neurobiology of craving in cannabis use disorder
Source: BMJ Open. 2025 Aug 31;15(8):e105854. doi: 10.1136/bmjopen-2025-105854 (PMC12406938; doi:10.1136/bmjopen-2025-105854)
Supplement: Supplementary file 3 [file bmjopen-2025-105854.reviewer_comments.pdf]

PEER REVIEW HISTORY

BMJ Open publishes all reviews undertaken for accepted manuscripts. Reviewers are asked to complete a checklist review form and are provided with free text boxes to elaborate on their assessment. These free text comments are reproduced below.

ARTICLE DETAILS

Title (Provisional)

CannChange: A protocol for a feasibility study using fMRI-based neurofeedback to change the neurobiology of craving in Cannabis Use Disorder

Authors

Murphy, Ethan; Dakhili, Amir Hossein; Ganesan, Saampras; Thomson, Hannah; Paloubis, Anastasia; Lorenzetti, Valentina; Kamboj, Sunjeev; Moffat, Bradford; Poudel, Govinda; Suo , Chao; Zalesky, Andrew; Glarin, Rebecca

---

VERSION 1 - REVIEW

**Reviewer**	**1**
**Name**	**Pongpirul, Krit**
**Affiliation**	**Chulalongkorn University Faculty of Medicine, Preventive and Social Medicine**
**Date**	**17-Jun-2025**
**COI**	**None**

I found this protocol an engaging and carefully constructed proposal for a first-in-field test of ultra-high-field (7 T) fMRI-neurofeedback in people with moderate-to-severe cannabis use disorder. The scientific premise is persuasive. By training participants to raise or lower activity in an individually localised region of the anterior cingulate cortex (ACC) while they view cannabis cues, the investigators address a well-established neural substrate of craving and relapse. The methods are explained in a level of detail that makes replication straightforward, and the research team has evidently listened to people with lived experience—changing “craving thermometer” to the more neutral “craving bar,” arranging weekend appointments, and offering meaningful reimbursement. These touches suggest a study culture that respects participants and maximises the chance of successful recruitment.

Before publication, however, a handful of clarifications would help readers judge whether the study ultimately achieves its principal aim: establishing feasibility. The manuscript would benefit first from a short paragraph that spells out what “feasible” will look like in concrete terms. For instance, if the team hopes to enrol and complete scanning for eight out of ten eligible volunteers within six months, to keep mean head motion below half a millimetre,

and to lose no more than two participants to attrition, stating these thresholds now will allow the eventual report to be interpreted at a glance.

A second issue concerns placebo and expectancy effects. Because the design does not include a sham or yoked-feedback control, any observed change in craving—or even ACC activity—could be driven partly by participants' expectations or by the novelty of lying in a 7 T scanner. A brief acknowledgement of this limitation, together with a sketch of how a sham condition might be engineered in a future, fully powered trial, would strengthen the discussion and forestall predictable reviewer queries at the next stage.

Related to transferability, the current protocol ends as soon as the final down-regulation run is finished. Even a five-minute "feedback-off" run, or a follow-up craving rating collected online the next day, would give a first hint of whether regulation skills generalise beyond the scanner environment. If time or budget prevents that addition in this feasibility round, it would still help to note explicitly that durability remains an open question to be tackled later.

The choice of a ten-person sample is typical for feasibility work, yet a sentence citing published guidance on sample size for pilot studies would reassure readers that the number is not arbitrary. References such as Julious (2005) or Sim and Lewis (2012) provide succinct justifications and could be slipped into the "Sample size" subsection without disrupting its flow.

Participant burden is another area worth a few extra words. A single session lasting four to five hours can be fatiguing, especially for individuals who may arrive with baseline anxiety or attentional fluctuation. Describing planned rest breaks, refreshments, or even the option to spread questionnaires over a second visit would show that practical welfare has been considered alongside methodological rigour. In the same vein, it would be prudent to outline how the team will respond if a participant's in-scanner craving or anxiety spikes unexpectedly—say, to a rating of nine or ten on the visual-analogue scale. A sentence noting that the scan can be paused, grounding techniques applied, and clinical referral offered if needed would cover this point.

A few minor housekeeping matters remain. Figure 1 could be even more useful if approximate durations for each task segment were added beside the arrows, giving readers an instant sense of pacing. The text lists certain questionnaires, such as the CUDIT-R and the Marijuana Ladder, at both screening and baseline; a brief explanation that the first appearance serves eligibility and the second provides a clean baseline would remove any impression of redundancy. Finally, the authors should confirm in the cover letter that the two instructional videos and Supplementary Sections 1.1 and 1.2 will travel with the submission, and they might run a last proof-read to catch a handful of rendering glitches (for example, a missing delta in "Δ^9-THC").

With these clarifications in place, the manuscript will stand as a transparent and methodologically sound foundation for the next phase of work. It already exhibits commendable care in design and respect for participants; refining the points above will

make the eventual feasibility results easier to interpret and will smooth the path toward a future randomised, controlled trial. On that basis I recommend acceptance after minor revision.

VERSION 1 - AUTHOR RESPONSE

Reviewer: 1

1. ***I found this protocol an engaging and carefully constructed proposal for a first-in-field test of ultra-high-field (7 T) fMRI-neurofeedback in people with moderate-to-severe cannabis use disorder. The scientific premise is persuasive. By training participants to raise or lower activity in an individually localised region of the anterior cingulate cortex (ACC) while they view cannabis cues, the investigators address a well-established neural substrate of craving and relapse. The methods are explained in a level of detail that makes replication straightforward, and the research team has evidently listened to people with lived experience—changing "craving thermometer" to the more neutral "craving bar," arranging weekend appointments, and offering meaningful reimbursement. These touches suggest a study culture that respects participants and maximises the chance of successful recruitment.***

Thank you for reviewing the manuscript and for acknowledging the novelty of the study and the strength of the methodology. We have carefully reviewed all the points raised and hope to have addressed these to your satisfaction.

2. ***Before publication, however, a handful of clarifications would help readers judge whether the study ultimately achieves its principal aim: establishing feasibility. The manuscript would benefit first from a short paragraph that spells out what "feasible" will look like in concrete terms. For instance, if the team hopes to enrol and complete scanning for eight out of ten eligible volunteers within six months, to keep mean head motion below half a millimetre, and to lose no more than two participants to attrition, stating these thresholds now will allow the eventual report to be interpreted at a glance.***
   Thank you for the note. We agree that we could have been more explicit about what "feasible' looks like for this study.

We have now included the below text in the Study Design section, page 9:
"The study will be deemed feasible if the target sample (n = 10) is recruited, screened, and undergoes the entire testing protocol."

3. ***A second issue concerns placebo and expectancy effects. Because the design does not include a sham or yoked-feedback control, any observed change in craving—or even ACC activity—could be driven partly by participants' expectations or by the novelty of lying in a 7 T scanner. A brief acknowledgement of this limitation, together with a sketch of how a sham condition might be engineered in a future, fully powered trial, would strengthen the discussion and forestall predictable reviewer queries at the next stage.***

Thank you for the note. We agree that a sham neurofeedback/placebo-control condition would be valuable. We have now included this in the 'Limitations' subsection and have detailed a potential procedure to decide on a control condition, page 30:

“Similarly, the primary objective of this study is to investigate if fMRI-neurofeedback can change craving-related brain activity in cannabis users. However, the lack of a sham/mock fMRI-neurofeedback control group means that we cannot confirm whether the effects reported herein are specific to fMRI-neurofeedback or non-specific neurobehavioral changes related to brain training, or from being in the scanner [71]. We recommend that future studies include an active-control group to confirm that the observed neural effects are specific to fMRI-neurofeedback and do not relate to more general processes. For instance, a cross-over design in a larger sample, where two intervention conditions (e.g., neurofeedback, mock) are administered to two carefully matched participant groups in a counterbalanced order, is warranted. Further, the mock neurofeedback condition should not be related to either visual-cue processing in substance users [18] or self-regulation processes underlying fMRI-neurofeedback training [73].”

4. ***Related to transferability, the current protocol ends as soon as the final down-regulation run is finished. Even a five-minute “feedback-off” run, or a follow-up craving rating collected online the next day, would give a first hint of whether regulation skills generalise beyond the scanner environment. If time or budget prevents that addition in this feasibility round, it would still help to note explicitly that durability remains an open question to be tackled later.***

We agree that a transfer run to assess the potential generalisable effects of neurofeedback would be valuable. The scope of this study was to test if the neurofeedback technical setup was actually technically feasible and adequate to measure and induce brain changes. We noted this in the ‘Limitations’ section, page 30:

“Fourth, the absence of a transfer run, and behavioural testing after the training day, limits our ability to investigate the transferability of the neurofeedback-learned craving regulation techniques to a non-neurofeedback context. Replication studies should integrate a transfer run and behavioural follow-ups to test these notions.”

5. ***The choice of a ten-person sample is typical for feasibility work, yet a sentence citing published guidance on sample size for pilot studies would reassure readers that the number is not arbitrary. References such as Julious (2005) or Sim and Lewis (2012) provide succinct justifications and could be slipped into the “Sample size” subsection without disrupting its flow.***
   Thank you for the note. We have now included the suggested references in the “Sample size” subsection, page 11:

“The target sample of 10 participants with moderate-to-severe CUD is due to the pragmatics of testing the feasibility of fMRI-neurofeedback. The target sample size equates to that recommended sample size for pilot studies [29]. The results will be used to inform power analyses and sample size estimations for larger-scale studies [30,31].”

6. ***Participant burden is another area worth a few extra words. A single session lasting four to five hours can be fatiguing, especially for individuals who may arrive with baseline anxiety or attentional fluctuation. Describing planned rest breaks, refreshments, or even the option to spread questionnaires over a second visit would***

show that practical welfare has been considered alongside methodological rigour. In the same vein, it would be prudent to outline how the team will respond if a participant's in-scanner craving or anxiety spikes unexpectedly—say, to a rating of nine or ten on the visual-analogue scale. A sentence noting that the scan can be paused, grounding techniques applied, and clinical referral offered if needed would cover this point.

Thank you for the note. We agree that fatigue and overwhelming feelings of craving could, in theory, present as potential issues for participants. We have now added a section in the Ethics and Dissemination section, page 28:

"Participant fatigue and emotional strain

An assessment session of between four to five hours could be fatiguing for participants, especially those who frequently use substances and who may arrive with elevated baseline anxiety [67, 68]. To reduce the effects of fatigue, all participants will be given the opportunity to have breaks at various points before and after MRI scanning; and will be provided with light refreshments. Similarly, several systems need to be in place should participants experience discomfort with any part of testing, including but not limited to craving and anxiety: researchers will offer to pause or cease the session and remind participants they can leave voluntarily without any consequences for their relationship with the team. Trained testers will be trained in techniques to support participants in these instances, such as compassionate communication and brief grounding techniques (e.g., grounding/progressive muscle relaxation) and mental health aid. A trained clinician will be made available for every testing session to support the tester in managing participants' anxiety/craving. Trained testers will also inform participants of free services with 24/7 availability at the testing location (e.g., Lifeline and Beyond Blue), the details of which will be given in the PIL and debrief forms."

7. ***A few minor housekeeping matters remain. Figure 1 could be even more useful if approximate durations for each task segment were added beside the arrows, giving readers an instant sense of pacing. The text lists certain questionnaires, such as the CUDIT-R and the Marijuana Ladder, at both screening and baseline; a brief explanation that the first appearance serves eligibility and the second provides a clean baseline would remove any impression of redundancy.*** Thank you for the note. We have now updated Figure 1 to reflect the suggested changes. We have now also added to the text outlining the screening process, page 13:

"Several metrics were administered at different stages of the testing protocol to serve distinct purposes: during online screening to ascertain study eligibility, and during face-to-face testing to measure key variables relevant for describing the sample and interpreting the data (e.g., substance use and related problems as assessed via the CUDIT and AUDIT)."

8. ***Finally, the authors should confirm in the cover letter that the two instructional videos and Supplementary Sections 1.1 and 1.2 will travel with the submission, and they might run a last proof-read to catch a handful of rendering glitches (for example, a missing delta in "Δ^9-THC").***

Once the proofs have been provided, we will be sure to run a last-proof read to catch any potential glitches and to confirm that the videos in the supplementary travel during submission.

9. ***With these clarifications in place, the manuscript will stand as a transparent and methodologically sound foundation for the next phase of work. It already exhibits***

commendable care in design and respect for participants; refining the points above will make the eventual feasibility results easier to interpret and will smooth the path toward a future randomised, controlled trial. On that basis, I recommend acceptance after minor revision.

Thank you again for reviewing the manuscript and for your appraisal of the quality of the work. We hope to have addressed your comments to your satisfaction.
